# Geometric Accuracy and Dimensional Precision in 3D Printing-Based Gear Manufacturing: A Study on Interchangeability and Forming Precision

**DOI:** 10.3390/polym17030416

**Published:** 2025-02-04

**Authors:** Xiaofeng Wei, Siwei Zhang, Lingli Sun, Xinyu Zhao, Mengchen Sun, Run Yu, Xingwen Zhou, Yuhang Li

**Affiliations:** 1 Institute of Spacecraft Application System Engineering, China Academy of Space Technology, Beijing 100094, China; 2 Microelectronics Instruments and Equipment R&D Center, Institute of Microelectronics of the Chinese Academy of Sciences, Beijing 100029, China; 3 School of Mechanical and Electrical Engineering, Soochow University, Suzhou 215000, China; 4 AVIC Research Institute for Special Structures of Aeronautical Composites, Jinan 250023, China; 5 College of Information and Communication Engineering, Harbin Engineering University, Harbin 150001, China

**Keywords:** 3D printing, fused deposition modeling, geometric tolerance, interchangeability, forming precision

## Abstract

This paper investigates the geometric interchangeability and dimensional precision of parts fabricated using Fused Deposition Modeling (FDM), with a focus on gear manufacturing. By employing a substrate and two spur gears as test components, critical process parameters, including layer thickness, extrusion speed, and print temperature, were optimized to achieve enhanced accuracy. Geometric and dimensional tolerances such as straightness, roundness, and surface roughness were systematically evaluated using advanced metrological techniques. The results indicate that larger components demonstrate higher precision, with deviations for large and pinion gears ranging between −0.045 and 0.060 mm, and −0.150 and 0.078 mm, respectively. Analysis reveals that the anisotropic nature of the FDM process and thermal shrinkage significantly impact accuracy, particularly in smaller features. Residual stress analysis reveals that smaller components formed via FDM exhibit higher stress concentrations and dimensional deviations due to voids and uneven thermal contraction, whereas larger components and flat substrates achieve better stress distribution and precision. The findings suggest that reducing material shrinkage coefficients and optimizing process parameters can enhance part quality, achieving dimensional tolerances within ±0.1 mm and geometric consistency suitable for practical applications. This research highlights the potential of FDM for precision manufacturing and provides insights into improving its performance for high-demand industrial applications.

## 1. Introduction

In recent years, with growing demand for complex components and high-performance materials in advanced fields such as aerospace, automotive manufacturing, and medical devices, 3D printing technology, particularly Fused Deposition Modeling (FDM), has found widespread applications [1,2,3]. As one of the most mature and cost-effective processes in the field of Additive Manufacturing (AM), FDM has gained significant attention for its ability to rapidly produce complex geometries while minimizing material waste [4,5]. However, in practical production and applications, FDM faces a major challenge: its geometric interchangeability and geometric accuracy often fall short of the stringent requirements of industrial manufacturing [6,7]. Geometric interchangeability refers to the ability of parts to be exchanged or replaced with others of the same specifications without compromising overall performance, while geometric accuracy encompasses the control of deviations in dimensions, shapes, and surface characteristics [5,8]. Key performance indicators for geometric accuracy typically include dimensional deviation, geometric tolerance, and surface roughness (*R_a_*) [9].

In traditional manufacturing, geometric interchangeability and accuracy can be achieved through high-precision machining processes and complex inspection equipment, but this often comes with high costs and low flexibility [10,11]. Although FDM technology shows significant advantages in manufacturing complex-shaped parts, it still has obvious shortcomings in manufacturing accuracy and interchangeability, due to multiple factors such as material properties, process parameters, and post-processing methods [6,12]. For example, due to the limitations of the layered processing principle, FDM parts often exhibit excessive dimensional deviations (usually over ±0.2 mm), insufficient interlayer bonding strength, and high surface roughness (*R_a_* values typically between 5 and 15 μm) [7,13]. These defects not only reduce the compatibility of parts in mechanical assembly, but may also significantly decrease the service life and performance of the parts, posing great difficulties for practical applications. Especially in scenarios requiring high-precision assembly (such as medical implants or aircraft engine components), any slight dimensional deviation can lead to irreversible consequences [3,14]. Therefore, how to improve the geometric interchangeability and accuracy of FDM technology by optimizing process parameters and material properties has become an important topic of common concern in both academia and industry [15,16].

In order to address the aforementioned issues, recent research efforts have focused on optimizing the key parameters of the FDM process to improve the geometric performance of parts. For example, parameters such as printing speed, layer thickness, nozzle temperature, and cooling rate have a direct impact on the dimensional accuracy and surface quality of the parts [4,7,17]. At the same time, material properties (such as thermal expansion coefficient and viscosity) and printing path design also significantly affect the geometric stability and interchangeability of the parts [11,18]. Existing studies have shown that by precisely controlling layer thickness and printing path, dimensional deviations can be controlled within ±0.1 mm; while optimizing nozzle temperature, layer thickness and material characteristics can reduce surface roughness *R_a_* values to 2–5 μm [9,19,20]. However, these optimization methods are mostly limited to specific materials or equipment, lacking widely applicable process optimization guidelines. Furthermore, current research rarely conducts a systematic evaluation of the performance of FDM parts from the perspective of geometric interchangeability, especially regarding the consistency of multiple prints and the stability of parts during long-term use [18,21,22]. Therefore, this study will systematically investigate the geometric accuracy and interchangeability of the FDM process, and propose a set of optimization strategies that are suitable for industrial production.

In this study, we aim to address the challenges of geometric accuracy and interchangeability in FDM-manufactured parts by systematically analyzing the key factors influencing dimensional precision and geometric tolerances. Specifically, we focus on the fabrication of spur gears and substrates, which are critical components in mechanical systems requiring high precision and interchangeability. Through a combination of experimental investigations and metrological analyses, we optimize key process parameters, such as layer thickness, extrusion speed, and print temperature, to improve the dimensional accuracy and assembly performance of FDM components. This work not only provides a detailed evaluation of the dimensional deviations and geometric characteristics of FDM-printed parts, but also explores the underlying mechanisms of these deviations, including thermal expansion, material shrinkage, and anisotropic deposition effects.

## 2. Experimental Materials and Methods

**Figure 1:**
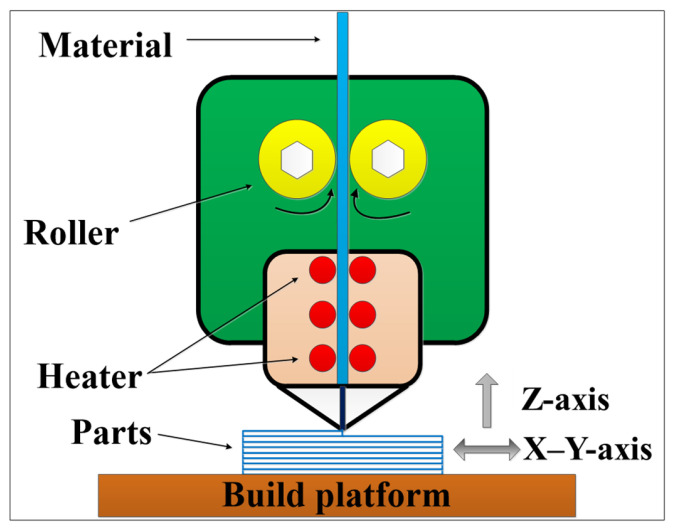
FDM process principle.

**Figure 2:**
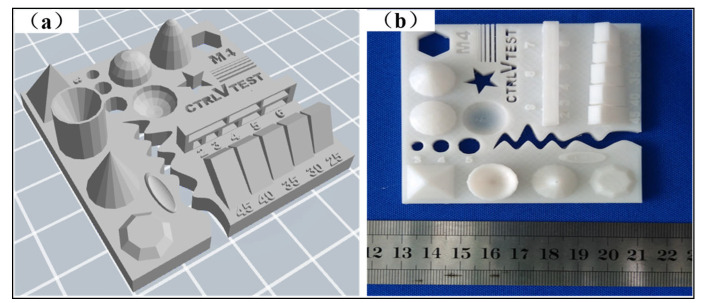
Reference model: (**a**) CAD model; (**b**) FDM-formed object.

The FDM process was performed using the MakerBot^®^ Replicator™ 2X 3D printer, manufactured by MakerBot (Brooklyn, NY, USA). The working principle of the FDM process is illustrated in Figure 1. Polylactic acid (PLA) polymer (model: 6252D, density: 1.24 × 10^3^ kg/m^3^) supplied by NatureWorks (Plymouth, MN, USA) was selected as the printing material. The mechanical properties of PLA materials manufactured by FDM can be seen in Table 1 [18]. To ensure the forming accuracy of the 3D printer and eliminate the influence of manufacturing and modeling errors during the process, a reference model, as shown in Figure 2, was designed. The reference model includes various geometrical features, such as planes, cylinders, spheres, cones, concave surfaces, convex surfaces, and overhanging structures. To ensure dimensional and geometric tolerances, features such as roundness, straightness, flatness, cylindricity, coaxiality, parallelism, perpendicularity, and inclination were controlled within acceptable error limits. Specifically, the actual printed dimensions were required to be no less than 95% of the design dimensions (CAD model) [4]. The percentage deviation (dimensional error) was calculated using Equation (1). This approach allowed for the systematic evaluation of the printer’s geometric accuracy and its capability to produce parts within the desired tolerance range [23].

(1) $$Percentage\;deviation=\left|\frac{designed\;\left(CAD\right)\;dimension-experimental\;dimension}{designed\;\left(CAD\right)\;dimension}\right|\times 100$$

**Figure 3:**
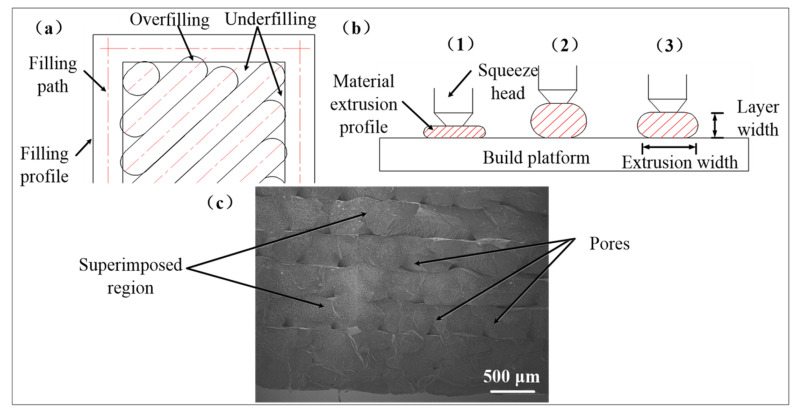
(**a**) Filament extrusion paths; (**b**) filament extrusion shape; (**c**) SEM morphology of infill pores.

To improve the dimensional accuracy and interchangeability of FDM parts, it is crucial to analyze the influencing factors in the manufacturing process, which are primarily divided into subjective (e.g., operational settings) and objective (e.g., material quality) factors [24]. Since objective factors are difficult to modify, optimizing subjective parameters becomes essential. Key issues include overfilling or underfilling areas caused by improper filling and extrusion speeds, as shown in Figure 3a. Additionally, nozzle height and filling speed significantly affect the extrusion shape, with extrusion thickness and layer width requiring precise adjustment of the distance between the printing platform and the nozzle, as depicted in Figure 3b. Achieving a saturated infill morphology minimizes pore size and quantity while preventing localized defects, as demonstrated in Figure 3c. Furthermore, suspended geometric features often require support structures, but their removal can deform the part and reduce surface accuracy and interchangeability [25]. To address this, this study avoids using support structures wherever possible, to enhance forming precision and performance.

Based on the above analysis, the filament diameter (1.75 ± 0.1 mm) and nozzle diameter (0.4 mm) were fixed parameters of the printer during the FDM process. To ensure the printed parts achieved optimal mechanical properties, the infill density and infill pattern were set to 100% and ±45°, respectively. The remaining parameters were systematically varied through combinations: three layer thicknesses (0.12 mm, 0.17 mm, and 0.25 mm), three extrusion speeds (35, 55, and 75 mm/s), three print speeds (35, 55, and 75 mm/s), and three printing temperatures (200, 210, and 220 °C). These parameters were selected based on common practices in FDM 3D printing, and to cover a range of typical operating conditions. The chosen layer thicknesses represent fine (0.12 mm), medium (0.17 mm), and coarse (0.25 mm) resolutions, allowing for the evaluation of surface quality and dimensional accuracy across different layer resolutions. The extrusion and print speeds (35, 55, and 75 mm/s) were selected to investigate their effects on material flow and layer bonding, with low, medium, and high speeds covering a broad range of printing conditions. The printing temperatures (200 °C, 210 °C, and 220 °C) were selected within the recommended processing range for PLA to study how slight variations in temperature influence material flow, bonding, and surface finish [3,13,21]. Therefore, these parameters were applied to form the reference models, and the optimal set of parameters, shown in Table 2, was determined using Equation (1). This parameter set was selected to facilitate the subsequent study of surface accuracy and assembly interchangeability of FDM parts. Using the optimal parameters from Table 2, five sets of samples were printed, with each set consisting of one base plate and two cylindrical gears. After printing, the samples were left to rest for 1–2 h. The geometric features of the base plate and two cylindrical gears, including straightness, flatness, roundness, cylindricity, parallelism, perpendicularity, inclination, positional tolerance, and coaxiality, were measured using the Mahr Surf XC20 profilometer (Mahr, Nuremberg, Germany) and Mahr MMQ 200 roundness gauge (Mahr, Nuremberg, Germany). Surface roughness was measured using the Taylor Hobson Surtronic 25 surface profilometer (Taylor Hobson, Leicester, Britain). The porosity and interlayer gaps of the printed parts were observed using the Hitachi S4800 scanning electron microscope (SEM, Hitachi High-Tech Corporation, Tokyo, Japan). Dimensional accuracy, positional accuracy, geometric accuracy, and contour accuracy of the printed parts were measured using the Hexagon 4.5.4 SF Coordinate Measuring Machine (CMM, Hexagon, Uppsala, Sweden) to study the forming precision and assembly interchangeability of the printed components. X-ray diffraction (XRD) was used to measure the residual stress distribution at the surface of the printed components. A Bruker D8 Advance X-ray diffractometer (Bruker, Billerica, MA, USA) was employed, and measurements were taken along multiple directions (X, Y, and Z axes) on the surface of both the main and pinion gears, as well as on the cylindrical surfaces of the substrate. The measurements followed the guidelines of ISO 1101:2017 [26] for the determination of residual stress in components. All residual stress measurements were performed after the FDM printing process was completed, and the parts were allowed to cool at room temperature for 1–2 h to minimize any immediate post-processing stresses. Residual stress testing was conducted under controlled conditions, with temperature and humidity monitored throughout the experiment to ensure consistency.

## 3. Experimental Results and Discussion

### 3.1. Establishment of the Geometric Model

**Figure 4:**
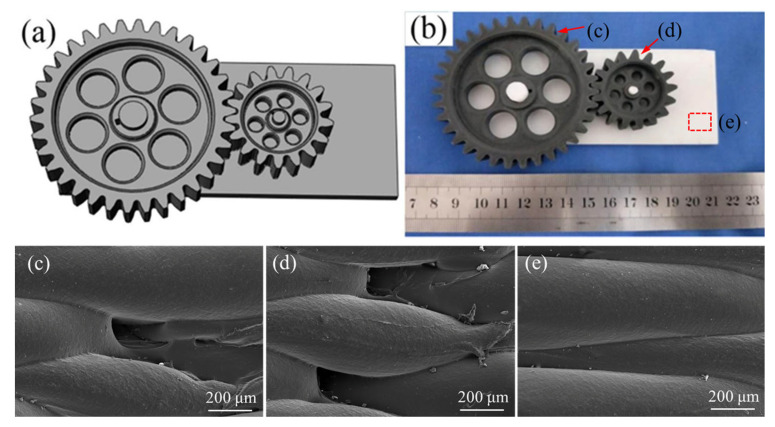
Gear assembly geometric model (including a substrate and two spur gears): (**a**) CAD model; (**b**) FDM formed object; (**c**–**e**) represent the SEM morphologies of the gear end face of the main gear, the gear end face of the pinion gear, and the upper surface of the substrate, respectively, in (**b**).

As a complex elastic mechanical system, gear transmission plays a critical role in engineering industries, where the design and manufacturing quality of gears directly impact the performance and reliability of mechanical products [27,28]. To investigate the interchangeability and forming precision of FDM technology, this study utilized a substrate and two spur gears as the primary test components, with the two gears printed separately to assess their compatibility and individual performance in the final assembly. The geometric model of the assembly is illustrated in Figure 4a,b. The design parameters for the gears and substrate are as follows:

Main gear: module *m* = 2, number of teeth *z* = 34, pressure angle *α* = 20°, coefficient of variation *χ* = 0, addendum diameter *Φ* = 72 mm, dedendum diameter *Φ* = 63 mm, tooth thickness 15 mm.

Pinion: module *m* = 2, number of teeth *z* = 17, pressure angle *α* = 20°, coefficient of variation *χ* = 0, addendum diameter *Φ* = 38 mm, dedendum diameter *Φ* = 29 mm, tooth thickness 15 mm.

Substrate: dimensions 120 × 50 × 5 mm, featuring two cylindrical pins with diameters: *Φ* 5 (height 18 mm) and *Φ* 10 (height 18 mm), spaced 51 mm apart.

Based on the comparison of the SEM morphologies in Figure 4c–e, it can be observed that the pinion gear’s gear end face exhibits the highest porosity, followed by the main gear’s gear end face, while the substrate surface shows almost no visible pores. The SEM image of the pinion gear (Figure 4d) reveals numerous small voids and gaps between the printed layers, indicating poor layer bonding and material flow inconsistencies during the FDM process. These porosities are likely a result of uneven cooling rates and material deposition, which is more pronounced in smaller components, due to the increased thermal gradients and the difficulties in achieving consistent extrusion at small scales [6]. In contrast, the main gear’s gear end face (Figure 4c) shows fewer surface defects, although some interlayer voids are still visible. The presence of fewer surface defects in the large gear can be attributed to its larger surface area and greater material deposition volume, which helps to mitigate some of the cooling and bonding issues seen in smaller components [16]. However, the SEM image still shows signs of incomplete layer bonding, indicating that while the large gear is less affected by porosity compared to the pinion gear, material deposition consistency remains an issue for both part sizes. Finally, the substrate’s upper surface (Figure 4e) exhibits a much smoother and more uniform surface, with virtually no visible pores. This indicates that the substrate, being a larger and simpler shape, experienced less thermal gradient and cooling issues during the printing process. The more consistent material flow and slower cooling rate for larger, flat surfaces result in a lower occurrence of porosity.

To enhance experimental diversity and ensure statistical reliability in testing, five identical sets of main gears, pinions, and substrates were fabricated using the FDM process. This approach facilitates the systematic evaluation of dimensional accuracy, geometric tolerances, and assembly interchangeability across multiple samples, providing comprehensive insight into the capabilities of FDM technology in producing complex mechanical components.

### 3.2. Dimensional Accuracy Inspection

**Figure 5:**
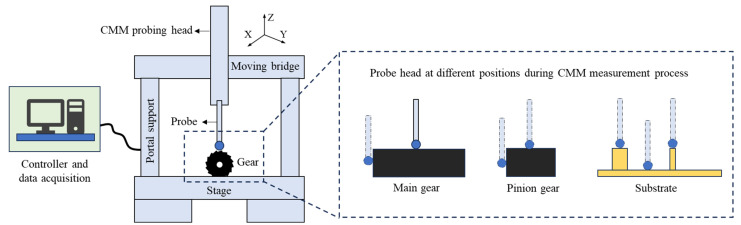
The schematic illustration of the measured features on the gears and the substrate.

**Figure 6:**
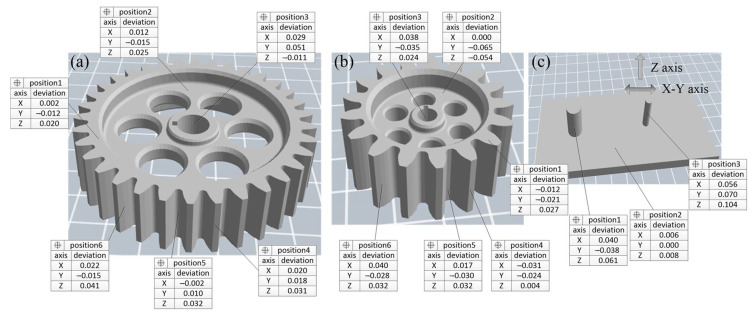
The dimension precision of the CMM measuring results: (**a**) main gear; (**b**) pinion gear; (**c**) substrate.

After forming, six measurement points were selected for both the main and pinion gear, including the gear plane, bearing surface, mounting hole, gear tooth tip, gear root, and gear flank. For the substrate, three measurement points were chosen: the large cylindrical surface, the small cylindrical surface, and the flat surface. At each measurement position, 10 points were measured using a Coordinate Measuring Machine (CMM), and the average value was calculated. The dimensional deviations in the X, Y, and Z coordinates, as well as the overall coordinate deviation, were controlled within ±0.1 mm. The schematic illustration of the measured features on the gears and the substrate can be seen in Figure 5, and the dimension precision of the CMM measuring results can be seen in Figure 6. From Figure 6, the main gear exhibits the highest precision, with deviations tightly controlled within a range of −0.015 mm to 0.051 mm. In contrast, the small gear shows a wider deviation range, from −0.065 mm to 0.040 mm, reflecting the challenges of maintaining accuracy in smaller geometries. The substrate demonstrates mixed results, with the large cylindrical surface achieving better precision (deviations within ~ ±0.04 mm) compared to the small cylindrical surface, which displays deviations ranging from −0.038 mm to 0.061 mm. These results suggest that larger features are less prone to thermal shrinkage and material flow inconsistencies, while smaller components are more affected by anisotropic effects and layer deposition inaccuracies.

**Figure 7:**
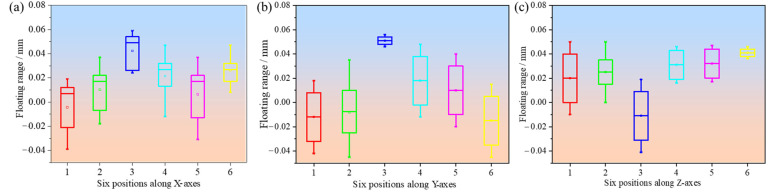
Dimensional deviations at six different positions along three different directions of the main gear: (**a**) X-axes; (**b**) Y-axes; (**c**) Z-axes.

**Figure 8:**
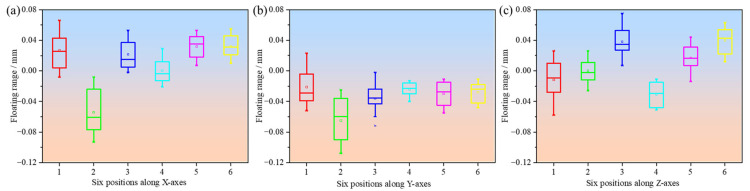
Dimensional deviations at six different positions along three different directions of the pinion gear: (**a**) X-axes; (**b**) Y-axes; (**c**) Z-axes.

**Figure 9:**
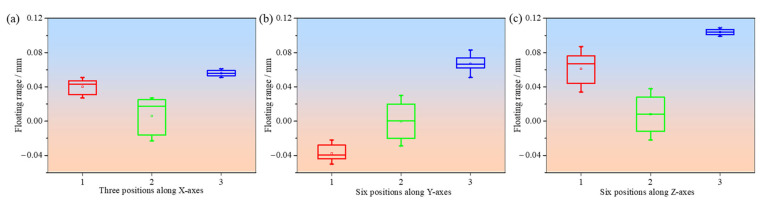
Dimensional deviations at three different positions along three different directions of the substrate: (**a**) X-axes; (**b**) Y-axes; (**c**) Z-axes.

Furthermore, the dimensional deviations for the five sets of samples at different measurement positions along the X, Y, and Z axes are shown in Figure 7, Figure 8, and Figure 9, respectively. For the main gear, the dimensional deviations range from −0.045 mm to 0.060 mm, as shown in Figure 7. These values are relatively small compared to the deviations observed in the pinion gear and substrate, indicating that larger components are generally more stable in terms of dimensional accuracy when produced through FDM. The deviation range observed in the main gear is tightly controlled, with the *X*-axis showing predominantly negative deviations, reflecting material shrinkage along this direction, while the Y- and Z-axes exhibit positive deviations, which may be attributed to slight expansion during the cooling phase. The dimensional accuracy of the main gear across different measurement points demonstrates the importance of part size in achieving precision. As noted, the deviations along the *X*-axis are mainly negative, with values ranging from −0.04 mm to −0.02 mm. In contrast, the deviations along the Y- and Z-axes are positive, ranging from 0.02 mm to 0.06 mm. This anisotropic behavior suggests that the cooling and solidification process during FDM is not uniform in all directions, leading to differences in dimensional stability [7]. The results confirm that for larger components, maintaining dimensional accuracy is less challenging compared to for smaller components. Additionally, the maximum comprehensive deviation (T) for the main gear is calculated as 0.068 mm, which is well within acceptable tolerance limits according to ISO 1101:2017, highlighting the effectiveness of the FDM process for producing reasonably accurate parts at larger scales.

In contrast, the small pinion gear shows significantly larger dimensional deviations, ranging from −0.150 m to 0.078 mm (Figure 8). These larger deviations are likely attributed to the inherent difficulties in producing smaller geometries with FDM. Small parts, such as the pinion gear, are more prone to dimensional inaccuracies due to several factors: inconsistent material flow can lead to uneven layer deposition, causing localized dimensional deviations; poor layer bonding introduces voids and weak interlayer adhesion, resulting in dimensional instability under mechanical or thermal stress; and greater susceptibility to thermal shrinkage and expansion, driven by higher thermal gradients during cooling, can cause warping and uneven contraction [29]. The results from the pinion gear measurements emphasize that as part size decreases, the precision of FDM-formed parts diminishes. This can be attributed to the limitations in extrusion control and the layer-by-layer deposition method of FDM, which struggles to maintain fine resolution in smaller structures [30]. A detailed comparison of the dimensional deviations for the main gear and pinion shows that the maximum comprehensive deviation (T) for the pinion gear is calculated as 0.119 mm, significantly larger than the value for the main gear (0.068 mm). According to the International standard ISO 1101:2017 [26], these deviations suggest that smaller components are more prone to errors during FDM fabrication. The increased deviations in the pinion may also be influenced by variations in cooling rates and material deposition consistency, which can disproportionately affect smaller geometries. As shown in Figure 8, deviations along the X- and Z-axes are predominantly positive for the pinion, with the largest deviation of 0.078 mm observed along the *Z*-axis at Position 3. The *Y*-axis shows mainly negative deviations, further emphasizing the anisotropic nature of the FDM process. These trends are consistent across multiple measurements, indicating that thermal and deposition effects contribute to dimensional inaccuracies in smaller components. This observation aligns with the general understanding that FDM-printed parts with smaller dimensions tend to exhibit reduced accuracy, particularly due to challenges in ensuring consistent material flow and precise layer alignment.

For the substrate, which consists of both large and small cylindrical features (Figure 9), the dimensional deviations are measured at three distinct positions: the large cylindrical surface, the small cylindrical surface, and the flat surface. The results indicate that the large cylindrical surface exhibits better dimensional accuracy, with deviations ranging from −0.06 mm to 0.10 mm. On the other hand, the small cylindrical surface shows a wider range of deviations, from −0.06 mm to 0.12 mm, emphasizing the increased challenges in maintaining dimensional accuracy for smaller features in FDM. This difference is particularly evident in the *Z*-axis, where the maximum deviation of 0.10 mm is observed for the small cylindrical surface. This further supports the observation that larger parts in FDM tend to achieve better precision, likely due to their increased stability during the printing and cooling processes [3]. The dimensional deviations of the substrate reveal the role that part size plays in the overall accuracy of FDM-printed components. As with the gear measurements, the *X*-axis deviations for the substrate are generally positive, indicating slight expansion, while deviations along the Y- and Z-axes are predominantly negative. The overall tolerance range for the substrate, which is between −0.06 mm and 0.10 mm, falls within acceptable limits for many industrial applications, but is still larger than the deviations observed for the main gear. This discrepancy highlights the impact of part size, thermal gradients, and material behavior on dimensional accuracy during FDM. As demonstrated in Figure 7, Figure 8 and Figure 9, the results consistently show that parts with smaller dimensions experience more significant deviations, which can be attributed to several factors, including the anisotropic nature of material deposition, variations in layer bonding strength, and the influence of cooling dynamics during the printing process [20,31]. These deviations are further compounded by the inherent limitations of the FDM process, such as inconsistent extrusion rates and the challenge of maintaining uniform material flow.

The analysis indicates that during the forming process, the majority of the material in the main gear exhibits shrinkage along the *X*-axis, while expansion dominates along the Y- and Z-axes. Conversely, for the pinion and substrate, most of the material shows shrinkage along the *Y*-axis, whereas expansion is more pronounced along the X- and Z-axes. This phenomenon can be attributed to the unique phase transition of PLA filament, which undergoes a solid–melt–solid transformation during the FDM process [4]. When PLA is melted, it experiences a rapid volumetric expansion due to the increase in temperature. However, during the subsequent solidification and cooling phase, the material undergoes shrinkage, leading to deviations in the final contour of the printed parts. These combined effects of thermal expansion and shrinkage contribute to the dimensional inaccuracies observed in the formed components [32,33]. Notably, the magnitude and direction of these dimensional changes are influenced by the orientation of the part on the build platform, the thermal gradients present during printing, and the anisotropic nature of material deposition inherent to the FDM process [7]. For smaller components, such as the pinion and the substrate’s small cylinder, the effects of thermal contraction are more pronounced due to the components’ reduced feature size, which amplifies the impact of material flow inconsistencies and local thermal gradients. These factors can result in more significant deviations and reduced dimensional accuracy for smaller geometries compared to larger ones.

### 3.3. Residual Stress Measurement

**Figure 10:**
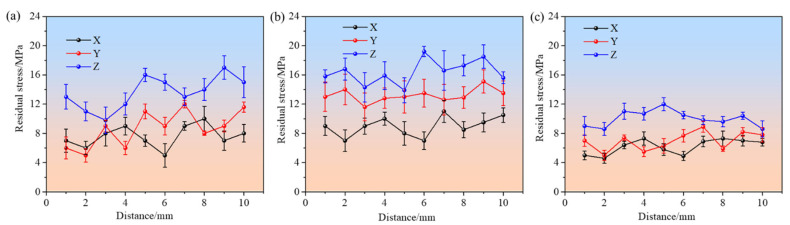
Residual stress test results of two spur gears and substrate along three directions: (**a**) main gear; (**b**) pinion gear; (**c**) substrate.

Based on Figure 10, the residual stress distributions for the main gear, pinion gear, and substrate along the X, Y, and Z directions exhibit significant variations, highlighting the influence of part geometry and the anisotropic nature of the FDM process. For the main gear (Figure 10a), the residual stress along the *X*-axis ranges from 5.1 MPa to 10.6 MPa, while along the *Y*-axis, it spans from 5.5 MPa to 12.6 MPa. The *Z*-axis residual stress demonstrates the highest variation, fluctuating between 9.8 MPa and 17.2 MPa. These differences can be attributed to the material shrinkage and thermal gradients during cooling, which are more pronounced in the vertical (Z) direction due to the layered deposition and heat accumulation effects [15]. For the pinion gear (Figure 10b), the residual stress shows a broader range of deviations. The *X*-axis stress ranges from 7.3 MPa to 10.6 MPa, the *Y*-axis from 11.6 MPa to 15.1 MPa, and the *Z*-axis from 13.9 MPa to 19.2 MPa. The larger residual stress values compared to the main gear are likely caused by the smaller geometry of the pinion, which results in increased thermal contraction and reduced layer stability. The smaller geometry leads to a higher surface-to-volume ratio, causing greater thermal gradients during cooling and exacerbating thermal contraction. Additionally, the reduced cross-sectional area in smaller components limits the stability of deposited layers, making them more prone to deformation and stress accumulation. Additionally, the smaller features of the pinion enhance the sensitivity to nozzle movement patterns and cooling dynamics, further contributing to the observed stress discrepancies. For the substrate (Figure 10c), the residual stress remains relatively uniform across the three axes. The *X*-axis ranges from 4.6 MPa to 7.3 MPa, the *Y*-axis from 5.5 MPa to 8.9 MPa, and the *Z*-axis from 8.6 MPa to 10.7 MPa. The uniform stress distribution is due to the flat geometry of the substrate, which facilitates even heat dissipation and minimizes anisotropic effects. The flat geometry provides a uniform surface area for heat transfer, allowing heat to dissipate more evenly across the part during printing and cooling. This reduces localized thermal gradients, which are typically responsible for stress concentrations, and ensures a more consistent cooling process. Moreover, the absence of intricate features reduces the impact of uneven material flow and localized thermal contractions.

To mitigate the effects of residual stress and dimensional deviations observed in FDM-formed parts, it is essential to optimize material properties. For instance, careful adjustment of process parameters, such as printing temperature, cooling rate, and nozzle movement speed, plays a pivotal role in minimizing thermal deformation. For applications requiring high precision, it is advisable to avoid using FDM for excessively small components, where dimensional accuracy and stress control are critical challenges due to the limitations of the process. The anisotropic nature of the FDM process arises from its layer-by-layer material deposition mechanism. Under computer control, the relative three-dimensional motion between the extrusion head and the forming platform creates precise deposition paths, as directed by the printing program [34]. After each layer is deposited, the extrusion head rises or the platform descends by a fixed height (i.e., the slicing layer thickness), continuing the process of scanning and filling until the entire solid part is formed. However, due to inherent process limitations, voids are often present between layers, as a result of either insufficient filling or overfilling. These voids can cause dimensional profile expansion or shrinkage, which in turn leads to deviations in the formed part. Additionally, the presence of voids introduces localized stress concentrations within the material [19,29]. During the heating and cooling cycles of the FDM process, these stress concentrations interact with the residual stresses generated by uneven thermal contraction. If the stresses exceed the material’s strain threshold, they can cause warpage deformation, leading to further dimensional inaccuracies. As highlighted in the residual stress analysis, smaller components, such as the pinion gear, are particularly susceptible to these effects, due to their intricate geometry and reduced structural stability. In contrast, larger components, such as the main gear, tend to exhibit better stress distribution, while the flat substrate demonstrates the least residual stress variation, due to its uniform geometry and more consistent thermal behavior.

### 3.4. Interchangeability Inspection

**Figure 11:**
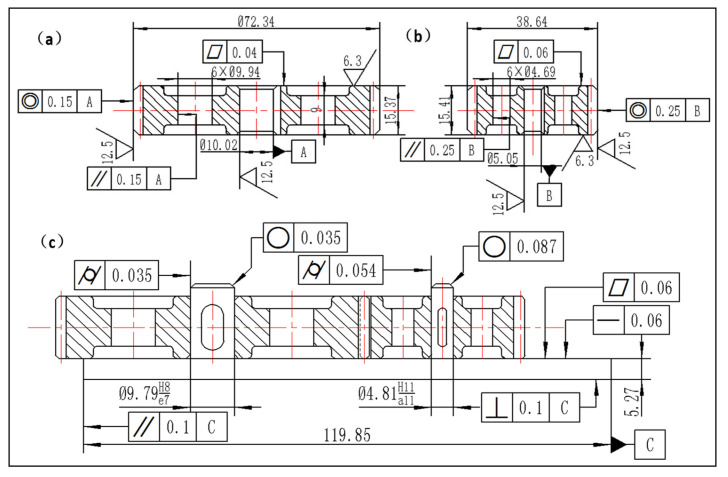
Interchangeability measurement results: (**a**) main gear; (**b**) pinion gear; (**c**) assembly mechanical drawing. In the figure, A represents the reference based on the mounting hole of the main gear; B represents the reference based on the mounting hole of the pinion gear; C represents the reference based on the horizontal surface of the substrate.

Interchangeability plays a crucial role in modern manufacturing, particularly in ensuring consistent assembly and performance of mechanical components [35,36]. To thoroughly evaluate the geometric interchangeability of the printed gears, comprehensive measurements of geometric characteristics were conducted, including straightness, flatness, roundness, cylindricity, parallelism, perpendicularity, and coaxiality. For statistical reliability, measurements were taken at five different positions for each geometric feature, and the average values were calculated. Additionally, basic dimensions and surface roughness of the formed parts were measured, as illustrated in Figure 11. Given that gear transmission requires relative motion during assembly, a clearance fit with a basic hole system was selected before mounting the gear pair on the base plate. As shown in Table 3, the mounting specifications were as follows: for the main gear mounting hole, the upper deviation was 0.022 mm and the lower deviation was 0 mm, while its shaft (large cylinder) had an upper deviation of −0.025 mm and a lower deviation of −0.04 mm. For the pinion mounting hole, the upper deviation was 0.075 mm and the lower deviation was 0 mm, with its shaft (small cylinder) having an upper deviation of −0.027 mm and a lower deviation of −0.345 mm. These specifications successfully met the assembly requirements. Analysis based on tolerance unit i, ISO Tolerance (IT) grades, and dimensional tolerance zones revealed that all geometric feature tolerances fell within the IT09–IT11 range, which aligns with the findings reported in [5,9]. Notably, Figure 11 demonstrates that for identical features, the main gear consistently achieved lower tolerance grades compared to the pinion, indicating superior precision in the main gear’s fabrication. A significant observation regarding surface quality showed that the roughness values were lower on the gear end faces compared to the gear tips. This phenomenon can be attributed to the minimal influence of the layer-wise printing structure on horizontal surfaces, whereas inclined and vertical surfaces exhibited more pronounced stair-stepping effects [28]. Consequently, the flatness of the formed parts demonstrated superior geometric tolerance compared to other features, while coaxiality characteristics showed the highest tolerance values. The dimensional deviation after formation was measured at 0.25 mm. This comprehensive geometric inspection validates the capability of FDM technology to produce interchangeable components within acceptable tolerance ranges.

**Figure 12:**
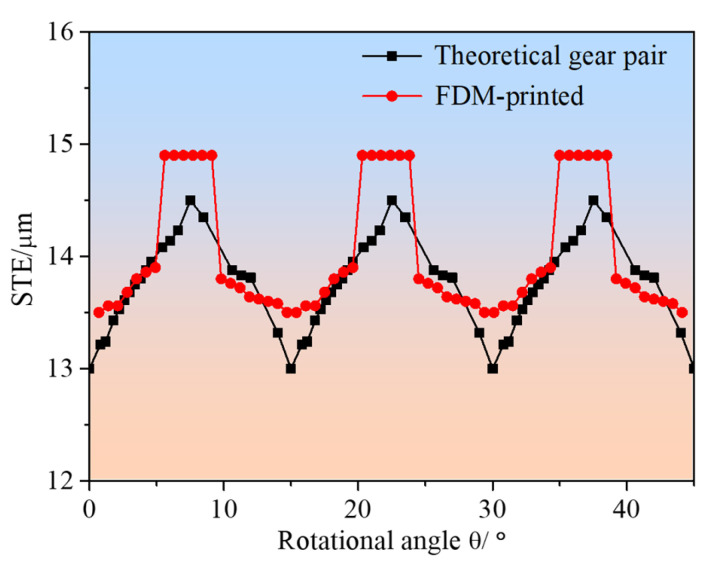
STE of gear pair.

Power transmission in gear systems occurs through meshing forces along the line of action, and inherent deviations between actual and theoretical tooth profiles are inevitable in any manufacturing process [35,37]. To validate this phenomenon in FDM-manufactured components, this study analyzed the Static Transmission Error (STE), a critical parameter that quantifies the integrated effect of various geometric deviations on gear mesh quality and transmission accuracy [38]. The STE curves for the gear pair are presented in Figure 12. Analysis of the results reveals that the theoretical gear pair exhibited a mean static transmission error of 13.82 μm, with a peak-to-peak variation of 1.52 μm. In comparison, the FDM-printed gear pair demonstrated a mean static transmission error of 14.05 μm, with a peak-to-peak variation of 1.41 μm. The remarkably small difference between these values—with variations contained within 5%—indicates that the FDM-manufactured gears maintain consistent mesh characteristics that are comparable to theoretical predictions. This stability in transmission error is particularly significant, because it suggests that despite the layer-by-layer manufacturing process inherent to FDM, the resulting gear pairs can achieve transmission accuracy suitable for practical applications. The consistency in STE values can be attributed to several factors in the FDM process: the precise control of material deposition, the optimization of printing parameters, and the careful consideration of gear geometry during the slicing process. These results demonstrate that when properly controlled, the FDM process can produce gear pairs with predictable and stable transmission characteristics, making them suitable for various mechanical power transmission applications where moderate precision is required.

## 4. Conclusions

This study systematically addresses the challenges of geometric accuracy and interchangeability in parts produced using Fused Deposition Modeling (FDM), focusing specifically on the fabrication of spur gears and substrates. The experimental results demonstrate that FDM can achieve reasonable dimensional precision and geometric accuracy, although there are inherent challenges such as thermal expansion, material shrinkage, and anisotropic deposition effects, particularly in smaller components. By optimizing key process parameters—including layer thickness, extrusion speed, and print temperature—we have significantly improved the dimensional accuracy and assembly performance of FDM parts. Our findings indicate that larger components tend to exhibit better accuracy, while smaller features are more susceptible to dimensional deviations, due to the limitations of material flow and layer bonding. The results highlight that careful control of printing parameters and material properties can mitigate the impact of these deviations, leading to more consistent and reliable part performance. Additionally, the study provides insights into the underlying mechanisms that contribute to dimensional inaccuracies, such as thermal contraction during cooling and variations in material deposition along different axes. Optimizing process parameters is essential to minimize void formation, residual stress accumulation, and warpage deformation, particularly for small and intricate FDM-printed components. These findings offer a comprehensive understanding of the factors that influence the precision and interchangeability of FDM components. Future research should focus on refining FDM for small, high-precision components, and developing more robust guidelines for process optimization to improve part consistency and long-term performance in industrial applications.

## Figures and Tables

**Table 1:** The mechanical properties of PLA materials manufactured by FDM technology.

Properties	PLA
Tensile strength (MPa)	15.5–72.2
Tensile modulus (GPa)	2.020–3.550
Elongation at break (%)	0.5–9.2
Flexural strength (MPa)	52–115.1
Flexural modulus (GPa)	2.392–4.930

**Table 2:** The optimal set of parameters.

Parameter	Value
Filament diameter	1.75 ± 0.1 mm
Nozzle diameter	0.4 mm
Object infill density	100%
Toolpath strategy	±45°
Layer thickness	0.12 mm
Deposition speed	35 mm/s
Feeding speed	55 mm/s
Print temperature	210 °C

**Table 3:** Deviation of basic bore system in clearance fit.

Deviation	Fit Hole of Main Gear	Fit Axle of Main Gear	Fit Hole of Pinion	Fit Axle of Pinion
Upper deviation	0.022	−0.025	0.075	−0.027
Lower deviation	0	−0.04	0	−0.345

## Data Availability

The raw data supporting the conclusions of this article will be made available by the authors on request.
